# Demystifying environmental health-related diseases: Using ICD codes to facilitate environmental health clinical referrals

**DOI:** 10.1177/18333583241300235

**Published:** 2024-11-26

**Authors:** Melissa Stoneham, Peter Schneider, James Dodds

**Affiliations:** 1 Curtin University, Australia; 2 Queensland Health, Australia; 3 Safe Food Production Queensland, Australia

**Keywords:** environment and public health, health policy, information management, health information management, classification, prevention and control

## Abstract

**Background:** The burden of disease of Aboriginal and Torres Strait Islander people is estimated as 2.3 times that of the broader Australian population, with between 30% and 50% of health inequalities attributable to poor environmental health. **Objective:** Although many Australian states and territories have clinical policy initiatives that seek to reduce the burden of preventable disease in this population, including field-based environmental health clinical referrals (EHCRs), there is little consistency across the jurisdictions, resulting in less potential to break the cycle of recurrent diseases within the home environment. **Method and Results:** This study addresses this inconsistency by recommending recognition and categorisation of environmental health risks to allow for accurate diagnosis and comparability across health services and locations by using the *International Statistical Classification of Diseases and Related Health Problems* (ICD) system, already in use in hospitals. **Conclusion and Implications:** Developing a list of mutually agreed environmental health attributable diseases for the EHCR process using assigned ICD-10-AM codes would influence the provision of primary care to include recognition of the impact of environmental health conditions and allow environmental health staff to provide a response and education at both community and household levels to break disease cycles.

## Introduction

It is estimated that 30%–50% of health inequalities experienced by Aboriginal and Torres Strait Islander peoples can be attributed to poor environmental health (Department of Health, 2013). The burden of disease of Aboriginal and Torres Strait Islander people is estimated as 2.3 times that of the broader Australian population (Australian Bureau of Statistics, 2023), and people living in remote communities’ experience hygiene-related diseases at rates higher than the wider Australian population (Foster and Dance, 2012; Hall, 2020). Many of these hygiene-related diseases are preventable and associated with the environmental health and housing conditions including overcrowding, non-functional health hardware and poor hygiene (Ali et al., 2018; Hall, 2020). Health hardware is the equipment inside a house that enables families to keep themselves healthy, such as functional showers, taps, toilets, stoves and refrigerators. Preventive health approaches around housing are predominantly enabled through environmental health initiatives conducted by local or regionally employed aboriginal environmental health practitioners (AEHPs).

Many Australian states and territories have clinical policy initiatives that seek to reduce the burden of preventable disease in Australian Indigenous populations. These include programmes for rheumatic heart disease, ear and eye health, and diabetes and renal health. These initiatives focus on clinical areas where Indigenous health outcomes are poorer, compared with the general population.

### Innovation position

This innovation position is contextualised in remote Australia and recognises and respects Aboriginal and Torres Strait Islander Australians who live on Country as custodians. It also acknowledges this as a human right under the United Nations Declaration on 136 the Rights of Indigenous Peoples (United Nations, 2007). Hereafter, the term ‘Indigenous’ is used to refer respectfully to Australia’s First Peoples, variously known as Aboriginal and Torres Strait Islander Peoples and First Nations Peoples (Australian Public Service Commission, 2022).

## The current study

**Figure 1.**
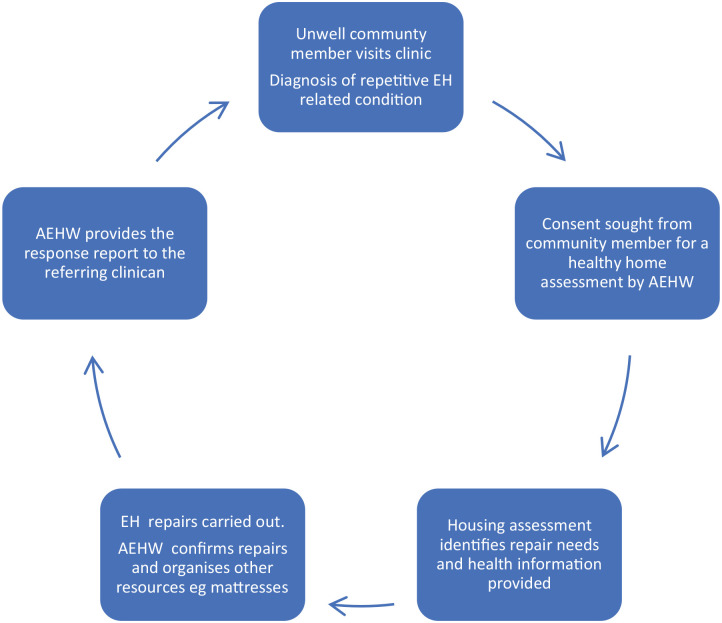
The environmental health clinical referral process.

In Australia, the environmental health clinical referral (EHCR) process has been developed in several state jurisdictions as a practical intervention in recognising and managing health hazards within Indigenous communities. EHCRs aim to establish a framework to ensure identification, treatment and prevention of recurring diseases that can be associated with the home environment. The process includes diagnosing the condition and linking it to risk factors within the home environment, providing targeted communication about the cause of the disease and its future implications, and asking the presenting community member to provide consent to enable an environmental health response (see Figure 1) to occur with the aim of reducing transmission or preventing recurrence within that home (Queensland Health, 2019; Stoneham, 2022). In general, the environmental health response aligns with the nine Healthy Living Practices, which describe the essential aspects required in a home to support personal health including the ability to wash people, wash clothes and bedding, remove waste safely, support nutrition, reduce crowding and reduce dust (Pholeros et al., 2013). An EHCR is a complex cyclical process as demonstrated in Figure 1.

### The environmental health clinical referral process

The importance of engaging with the home environment to reduce disease cannot be understated. In one study investigating otitis media (OM) in Indigenous children, housing-related determinants were reported almost three times more than the next most frequently reported risk factor (DeLacy et al., 2020). In this study, despite the acknowledgement of the association between housing and the prevalence of OM in Indigenous children, the authors could not find any intervention studies within the reviewed literature that investigated how to effectively address the issue of housing in Indigenous populations.

The EHCR process, if used in all remote clinics, has the potential to break the cycle of recurrent diseases within the home environment. Currently, the process of implementing EHCRs is not consistent or mandated. EHCRs are administered by clinics, some of which are Aboriginal controlled, and others are run through the state or regional health departments. Some agencies collect the data digitally, while others do not collect the data at all. Regions within the two jurisdictions all manipulate the process slightly, and the itemised diseases on the EHCR forms vary. All these issues make it very difficult to collect meaningful data or allow comparisons across regions. More specifically, in one state jurisdiction, the generic digital EHCR form does not currently provide a list of environmental health-related conditions for the clinician, but instead is an open-ended question for completion. In another state jurisdiction, a list of diseases is itemised; however, the list of diseases varies between regions. One area of consistency across both jurisdictions is that following the clinic visit, the follow-up EHCR activities may involve the following:

- Talking about the environment and how sickness spreads
- Providing information on how to stop sickness in the home
- Conducting a health hardware assessment of the living spaces
- Supporting the tenant to report house repairs and maintenance issues, and
- Getting back in touch with the clinic to advise on action and discuss ongoing health problems (Queensland Health, 2019; Western Australian Health, 2024).

These activities represent the cyclical nature of the EHCR process.

Without a list of agreed diseases to be included on the EHCR list, the consistency of interventions and effective policy for healthy housing cannot be achieved fully. Developing a list of mutually agreed environmental health attributable diseases for the EHCR process would influence the provision of primary care to include recognition of the impact of environmental health conditions and allow environmental health staff to provide a response and education, at both community and household levels to break disease cycles. A critical component of the EHCR process is the environmental health response, which is designed by the local AEHP following an assessment of the health hardware and home environment. As a community-based professional, local AEHPs provide a unique and culturally appropriate link between their communities and clinical services. It has been suggested that no other set of health professionals is as well placed to provide culturally appropriate environmental health or hygiene services to Indigenous people within their communities (Stoneham, 2020). Engaging community-based environmental health practitioners and recognising them as experts in their communities, is vital to ensure successful planning, development, implementation, and evaluation of the EHCR process within an Australian Indigenous context. It is critical that remote clinicians understand and accept the role that AEHPs play in community, and the benefits they can provide for health communication and community outcomes. A trained and supported AEHP can be a change agent for community and together with clinicians, can lead to improvements across health infrastructure and reduce the primordial drivers of poor health in their communities.

In the EHCR process, the environmental health response is designed to address the risk factors of the presenting condition, ensuring the response is tailored and meaningful. The response can vary from providing small plumbing fixes, to ensuring functional health hardware, through to the provision of mattresses, or clothes and blanket washing facilities.

For EHCRs to be effective, there must be a consistent recognition and categorisation of environmental health risks to allow for accurate diagnosis and comparability across health services and locations. One avenue to ensure consistency is by using the *International Statistical Classification of Diseases and Related Health Problems* (ICD) system, already in use in hospitals. The ICD classifies and codes diseases, signs and symptoms, abnormal findings, social factors and external causes of mortality or morbidity (Harrison et al., 2021; Independent Health and Aged Care Pricing Authority (IHACPA), n.d.). It is used globally by clinicians, clinical coders, policymakers and researchers and provides critical knowledge on the extent, causes and consequences of human disease and death worldwide via data that are reported and coded with the ICD (Australian Institute of Health and Welfare, 2023). In Australia, health conditions and injuries are assigned ICD-10-AM (Australian Modification) codes, resulting in data that can be used by governments to design effective public health policies, and measure their impact or used for clinical recording.

## What can be learned from this study?

### Linking EHCRs, remote housing and the ICD

The importance of living conditions, and specifically housing, has been recognised for centuries as a fundamental requirement for health (Hood, 2005). The link between hygiene practices, the home and health has been well-studied, and it is evident that addressing the home environment is fundamental to adequately managing many environmental health related conditions. Yet, the inadequacy of housing and housing maintenance for Indigenous Australians has been widely acknowledged (Bailie and Wayte, 2006; Stoneham, 2022).

Existing research indicates that housing conditions can influence a range of health conditions, all of which are preventable. For conditions that specifically relate to the home environment as discussed below, the evidence is clear that the following health conditions (ICD code shown in brackets) should be included on any EHCR form.

- *Gastrointestinal infections* (A00–A09) have been associated with poorly maintained housing and the state of food preparation and storage areas, the presence of mould and mildew, crowding, lack of infrastructure to wash people, clothes and bedding and safe removal of faecal matter (Bailie and Wayte, 2006). There is strong evidence that handwashing with soap can prevent diarrhoeal disease among children (McDonald et al., 2008).
- *Skin-related diseases* such as scabies (B86), impetigo, boils and general skin sores (L00–L08) have been associated with crowding (Ali et al., 2018; McDonald et al., 2008), poor living conditions (Foster and Hall, 2016) and the presence of pests and vermin (Ali et al., 2018). Handwashing with soap, functional health hardware and encouraging frequent bathing has shown to reduce impetigo (Gramp and Gramp, 2021; Luby et al., 2005).
- *Scabies* has specifically been associated with crowding within sleeping accommodations and sharing of clothes (Melese et al., 2023). Exposure to Group A Streptococci is also responsible for the continuing high rate of acute rheumatic fever (Katzenellenbogen et al., 2020) (I00–I02), rheumatic heart disease (I05–I09) and acute post-streptococcal glomerulonephritis (N00.1–9) among children. These diseases should be included on an EHCR referral form.
- *Viral conditions* such as influenza (J10–J11) have been associated with mould and mildew, generic housing and hygiene factors (Ali et al., 2018) and crowding (McDonald et al., 2008). Handwashing with soap, functional health hardware and encouraging frequent bathing have been associated with a lower incidence of pneumonia (J12–J18) (Ali et al., 2018; Luby et al., 2005).
- *Trachoma* (A71) has been associated with face washing (West, 1995), availability of soap, crowding within beds, having access to household sanitation (Stocks et al., 2014) and a lack of access to clean water (Warren and Birrell, 2016).
- *Otitis media* (H65–H67) has been associated with crowding (Bailie and Wayte, 2006; Jervis-Brady et al., 2014), a lack of functioning facilities for washing people and bedding and sewage removal (DeLacy et al., 2020). Other risk factors that can be addressed within the home environment include exposure to tobacco smoke and malnutrition (Jervis-Bardy et al., 2014). Although there are ICD-10 codes for some of the risk factors mentioned in the Jervis-Bardy study such as exposure to tobacco smoke (Z58.7), they are generally not very specific or related to housing issues.

In the revised ICD-11, one of the new core chapters is ‘Conditions related to sexual health’ (World Health Organization, 2019). To be contemporary and enhance the consistency of the EHCR process, it is recommended that a similar chapter be added on ‘Conditions related to environmental health’. This could be achieved in Australia by advocating for public submissions to the Independent Health and Aged Care Pricing Authority (IHACPA, 2023) to enable the collection of more specific information by having more specific codes. Adding additional codes, or a chapter that would integrate the diseases discussed above, would enable the acceptance, counting and identification of health issues related to remote housing. Policy could then be designed to reduce these conditions via an up-to-date and clinically relevant classification system.

## Conclusion

The risk factors for many environmental health-related diseases are found within the home environment. Addressing the home environment is consequently fundamental to adequately managing environmental health related diseases. The EHCR process, if implemented consistently across sectors and communities, has the potential to prevent these environmental health-related diseases early in life as well as break the cycle of disadvantage that contributes to social determinants driving ill-health across the life-course. For conditions that can specifically relate to risk factors in the home environment such as gastrointestinal infections, skin-related conditions, acute rheumatic fever, viral or respiratory illnesses, OM and trachoma, the ICD codes could be used to ensure a consistent EHCR process in the Australian context.
